# Nocturia: consequences, classification, and management

**DOI:** 10.12688/f1000research.11979.1

**Published:** 2017-09-01

**Authors:** Noam D. Fine, Jeffrey P. Weiss, Alan J. Wein

**Affiliations:** 1 Department of Urology, SUNY Downstate Medical Center, Brooklyn, New York, USA; 2 Division of Urology, University of Pennsylvania, Philadelphia, Pennsylvania , USA

**Keywords:** Nocturia, urology, overactive bladder

## Abstract

Nocturia is a widespread condition that can negatively impact quality of sleep and overall health. This condition is multifactorial in nature and is best approached through the analysis of frequency volume charts. Through these charts, clinicians may classify each individual case of nocturia into one of four distinct categories: global polyuria, nocturnal polyuria, reduced bladder capacity, and mixed. Treatments should then be tailored to each individual based upon the category of their nocturia. In some cases, appropriate therapy will consist of behavioral modification techniques or addressing underlying systemic diseases. In other cases, medical therapy may be necessary, but, to date, medications have shown limited efficacy at treating nocturia.

## Introduction

Nocturia is a prevalent and highly bothersome lower urinary tract symptom (LUTS). In 2002, the International Continence Society (ICS) defined nocturia as “the complaint that the individual has to wake at night one or more times to void” ^1^. There has since been much debate around this definition, and it was revised in 2017 to be “waking to pass urine during the main sleep period” ^2^. Part of the controversy over the original definition stemmed from the fact that there may be no “complaint” from those who wake only once during the night to void, as a single episode of nocturia may not be bothersome at all. The Finnish National Nocturia and Overactive Bladder (FINNO) study found that the majority of people report having at least moderate bother when they experience three or more nocturnal voids and that the degree of bother increases with the number of nightly voids ^3^. Furthermore, this study demonstrated that nocturia is associated with impaired health-related quality of life (HRQoL) as measured by the generic 15D HRQoL instrument ^4^. Unfortunately, many individuals do not seek medical consultation for nocturia because of the perception that it is a normal part of aging or even that it is untreatable ^5^.

This article aims to review the prevalence of nocturia along with its clinical consequences. A classification scheme for nocturia is presented and corresponding management strategies for each category are discussed.

## Epidemiology

Prevalence rates for nocturia vary by age and gender, but all populations can be affected ( Table 1) ^6^. For both genders, there is a tendency for nocturia to increase with age. Nevertheless, it is important to identify and treat younger individuals with nocturia, since the detrimental effects of sleep fragmentation are especially difficult for those with active lifestyles and demanding work schedules ^6^. A study in Finland reported higher rates of nocturia in women as compared to men for younger populations, but this gender difference disappeared at ages 50–59 and the prevalence became higher among men beyond the age of 60 ^7^.

**Table 1.** Prevalence of nocturia.

	Prevalence Rates	
	****
**Men**	*One or more* *void per night*	*Two or more* *voids per night*
20–40 years	11–35.2%	2–16%
>70 years	68.9–93%	29–59.3%
**Women**			
20–40 years	20.4–43.9%	4.4–18%
>70 years	74.1–77.1%	28.3–61.5%

## Consequences of nocturia

By definition, nocturia results in sleep interruption, and thus it is axiomatic that this condition is associated with lower sleep quality. A multivariate analysis of elderly individuals (55–84 years of age) interviewed by the National Sleep Foundation showed that nocturia was an independent predictor of self-reported insomnia and poor sleep quality ^8^. In a cross-sectional analysis of the Sleep Heart Health study, patients with nocturia reported higher degrees of subjective sleepiness and had corresponding polysomnographic changes that indicated objectively worse sleep quality ^9^. The first nocturnal void in particular has the potential to lower sleep quality, since it may occur during the first few hours of sleep, interrupting the restorative slow-wave sleep that takes place during this time ^10^.

Sleep interruption has numerous negative consequences for those with nocturia, including daytime fatigue, difficulty concentrating, mood alterations, and decreased workplace productivity ^11^. Falls and bone fractures have been reported to occur at higher rates among those with nocturia; these events may occur during the day because of fatigue caused by nocturia or during the night when individuals rise to void ^12^. Several studies have explored even more severe consequences of nocturia, and a meta-analysis of 28,366 patients found that nocturia was associated with a 28% excess mortality risk per year ^13^.

## Evaluation of nocturia

The evaluation of patients with nocturia begins with a detailed history that includes questions relevant to voiding behavior ^1, 2^. Underlying medical conditions that may account for increased voiding at night should be considered, and routine urine tests (i.e. dipstick or urinalysis) can be performed. Patients should also complete a frequency volume chart (FVC) in which they record the time and volume of each void over at least a 24-hour period. The FVC is a key diagnostic tool because it allows clinicians to classify each individual case of nocturia into one of four basic categories: global polyuria, nocturnal polyuria, reduced bladder capacity, and mixed. An overview of parameters that can be derived from the FVC is shown in Table 2.

**Table 2.** Parameters derived from frequency volume chart.

Parameter	Definition
NUV	Total nocturnal urine volume, including the first morning void
NPi	NUV / 24-hour urine volume
MVV	Maximum single voided volume over a 24-hour period
Ni	NUV/MVV
PNV	Ni – 1
ANV	Actual number of nocturnal voids
NBCi	ANV – PNV

MVV, maximum voided volume; NBCi, nocturnal bladder capacity index; Ni, nocturia index; NPi, nocturnal polyuria index; NUV, nocturnal urine volume; PNV, predicted number of nocturnal voids.

## Global polyuria

Global polyuria occurs when urine is produced in excess of 40 mL/kg bodyweight during a 24-hour period ^1^. Such urine overproduction may simply be the result of increased fluid intake, or it may be the manifestation of an underlying disease, often stemming from the renal or endocrine system ^14^. Examples of diseases causing global polyuria include poorly controlled diabetes mellitus, central or nephrogenic diabetes insipidus (DI), and primary polydipsia (psychogenic/behavioral or dipsogenic, latter due to brain trauma, radiation, or surgery with attendant dysfunctional thirst mechanism).

## Nocturnal polyuria

Nocturnal polyuria occurs when urine is overproduced at night while 24-hour urine production volume remains normal. The proportion of urine produced at night can be quantified using the nocturnal polyuria index (NPi), which is calculated as the nocturnal urine volume (NUV) divided by 24-hour urine volume. Importantly, the NUV is defined as the total volume of urine voided during the night, including the first morning void. The ICS defines nocturnal polyuria in an age-dependent manner as nocturnal output greater than 20% of the daily total in the young and greater than 33% of the daily total in the elderly (NP33) ^1^.

By the NP33 definition, nocturnal polyuria may be present in up to 83% of those with nocturia ^7^. Some have argued that this definition is too broad and lacking in clinical significance. In a meta-analysis of the difference in mean nocturnal voiding frequencies (NVFs) between patients with and without nocturnal polyuria, Hofmeester *et al*. found a mean difference in NVF of only 0.59 ^15^. Furthermore, a longitudinal, community-based study of 1,688 men aged 50–78 in Krimpen found that nocturnal polyuria was present in 70.1% of men without nocturia when the ICS definition was applied ^16^.

These limitations have led to several alternative definitions for nocturnal polyuria. van Haarst *et al*. suggested still defining nocturnal polyuria based upon the NPi but raising the threshold to 53%, which corresponds to the 95% of NPi values ^17^. Others have suggested definitions that move away from NPi entirely; in these cases, nocturnal polyuria may be defined as an NUV of >0.9 mL/minute, NUV of >6.4 mL/kg, or a nocturnal urine production rate of >90 mL/hour (NUP90) ^18^. The NUP90 cutoff is based on the finding that the mean hourly urine production rate during sleep in the Krimpen study was 60 mL/hour and two standard deviations above that mean value was 90 mL/hour. An advantage of the NUP90 definition of nocturnal polyuria is that it can be calculated in patients who have supplied nocturnal-only diaries ^19^.

As with global polyuria, nocturnal polyuria can be the result of specific behaviors (i.e. excessive nocturnal fluid intake) or the manifestation of underlying systemic disease. Obstructive sleep apnea (OSA) can lead to nocturnal polyuria owing to increased levels of circulating atrial natriuretic peptide (ANP), which promotes both water and osmotic diuresis. Peripheral edema for any reason—hepatic failure, nephrotic syndrome, or chronic venous insufficiency—can lead to nocturnal polyuria via third space fluid sequestration ^11^. This occurs when there is a net transfer of fluid from the extracellular (“third space”) compartment back into the intravascular compartment at night, which can provide an additional free water load that is removed by the kidneys. Those suffering from congestive heart failure (CHF) may experience nocturnal polyuria via a combination of these mechanisms, as they may have elevated levels of ANP and fluid sequestered in the third space.

## Reduced bladder capacity

Reduced bladder capacity occurs when there is discordance between urine production and the bladder’s ability to store urine, which can be global or nocturnal in nature. Reductions in nocturnal bladder capacity can be diagnosed through the nocturnal bladder capacity index (NBCi), where NBCi values above zero indicate that nocturia is occurring at voided volumes less than the MVV.

The pathophysiology of nocturia in patients with overactive bladder (OAB) or benign prostatic hyperplasia (BPH) may be the result of a reduced bladder capacity. In patients with OAB, detrusor overactivity can trigger the urge to void at volumes below the actual bladder capacity ^20^. A small but important study of nine patients with OAB and evidence of detrusor overactivity on cystometrogram found that these patients had a significantly greater mean number of nocturnal detrusor overactivity events as compared to controls and individuals with insomnia ^21^. This study was unique in demonstrating the relationship between detrusor contractions and transformation of the polysomnogram from the sleeping to the awake state.

BPH can reduce bladder capacity via a different mechanism, whereby patients are left with high post-void residual volumes owing to the inability to completely empty the bladder ^22^. A component of detrusor overactivity can be present in these patients when there is concomitant bladder outlet obstruction. If obstruction is left untreated or treated only with medical therapy, this overactivity may actually persist for long periods of time ^23^.

There are numerous other conditions, both urologic and non-urologic, that can lead to reduced bladder capacity ( Table 3) ^24^.

**Table 3.** Causes of reduced bladder capacity.

Bladder calculi Ureteral calculi Neoplasms of bladder, prostate, or urethra Anxiety disorders Learned voiding dysfunction Pharmacologic agents Neurogenic bladder Cystitis Extrinsic compression

## Mixed

For some patients, analysis of the FVC will reveal the etiology of their nocturia to be mixed. A retrospective study of 200 patients with nocturia found that 36% were classified as mixed on the basis of having both nocturnal polyuria and a reduced nocturnal bladder capacity ^25^.

## Management strategies

Behavioral modification techniques are appropriate first-line treatment for patients with nocturia, regardless of the etiology. These include modifying global and evening fluid intake, restricting the consumption of foods that promote diuresis, and improving patient understanding of the physiology of storing and voiding urine ^26^. Further interventions can be tailored to each patient based upon the pathophysiology of their nocturia.

### Global polyuria

The treatment for global polyuria will vary depending upon the underlying reason for increased urine production. Uncontrolled diabetes mellitus must be properly managed with appropriate lifestyle changes and medical therapy. Central DI can be treated with desmopressin, a vasopressin analog that promotes water reabsorption in the kidneys ^27^. High-dose tablet and low-dose sublingual forms of desmopressin have been found to reduce the mean number of nocturnal voids, even in patients without DI ^28^. Those with primary polydipsia may require psychotherapy in order to reduce their fluid intake ^22^.

### Nocturnal polyuria

Initial treatments for nocturnal polyuria should be directed at identifying and addressing any relevant systemic disease, such as CHF or OSA. In a meta-analysis of five studies, patients with OSA and nocturia had reductions in the frequency of nocturia and night-time urine volume after treatment with continuous positive airway pressure (CPAP) ^29^. In patients taking diuretics, the administration of these medications 6 hours prior to bedtime can reduce night-time frequency and the percentage of night-time voided volume ^30^. When peripheral edema is a concern, simple evening leg elevation or compression stockings can redistribute third space fluid ^9^. As mentioned above, desmopressin can reduce nocturia severity, but its major adverse effect is hyponatremia ^31^. Accordingly, serum sodium testing is advisable prior to beginning desmopressin therapy, especially for those over the age of 65, since these individuals are most at risk of developing hyponatremia ^32^. For the newly approved desmopressin acetate nasal spray, clinical trials reported serum sodium levels of 126–129 in 1.4% of cases at a dose of 0.75 μg and in 2.0% of cases at a dose of 1.5 μg ^33^.

### Reduced bladder capacity

Alpha-blockers are a central treatment for BPH, and a number of studies have explored their efficacy at treating associated nocturia. The Medical Therapy of Prostatic Symptoms trial found that doxazosin alone reduced the number of nightly voids by 0.54, only slightly outperforming placebo, which caused a reduction of 0.35 ^34^. This study also suggested that 5-alpha-reductase inhibitors offer only minimal improvement in nocturia, a finding that has been borne out in several other studies ^35, 36^.

Nocturia may improve following surgical interventions in those who have appropriate indications for such treatments. A prospective randomized study of 66 men with BPH and nocturia showed that transurethral resection of the prostate (TURP) reduced the mean number of nightly voids by one at 1-year follow-up, outperforming tamsulosin by 0.34 awakenings per night ^37^. Antunes *et al*. showed that the benefits of TURP at improving nocturia are present even in cases where the amount of resected tissue is less than 30% ^38^. Despite these findings, a study of 104 men who underwent radical prostatectomy found improvements following surgery in all domains measured by the American Urological Association symptom index, except for nocturia ^39^.

OAB medications appear to have minimal effect at treating nocturia. A meta-analysis comparing the efficacy of various antimuscarinics for those with OAB and nocturia found trospium chloride to be most effective, yet this medication reduced the mean number of nocturia episodes by only 0.24 compared with placebo ^40^. A separate study found that mirabegron reduced the mean number of nocturnal voids by only 0.39 compared to placebo ^41^. The clinical significance of OAB medications is therefore likely limited for treating nocturia.

## Conclusion

Nocturia is a prevalent condition that severely impacts quality of life for many individuals. It is important to recognize nocturia as an entity in its own right, since it is often misunderstood and, at times, even left untreated ^3^. Nocturia is multifactorial in nature, so no single treatment will prove effective for all patients. Instead, treatments should be tailored to individuals based upon the type of nocturia revealed from FVC analysis.
